# Transmission Dynamics of Bovine Viral Diarrhea Virus in Hokkaido, Japan by Phylogenetic and Epidemiological Network Approaches

**DOI:** 10.3390/pathogens10080922

**Published:** 2021-07-21

**Authors:** Shizuka Hirose, Kosuke Notsu, Satoshi Ito, Yoshihiro Sakoda, Norikazu Isoda

**Affiliations:** 1 Laboratory of Microbiology, Department of Disease Control, Faculty of Veterinary Medicine, Hokkaido University, Kita 18, Nishi 9, Kita-ku, Sapporo 060-0818, Hokkaido, Japan; shizuka-1612asteroid@eis.hokudai.ac.jp (S.H.); kousuke_notsu@med.miyazaki-u.ac.jp (K.N.); sakoda@vetmed.hokudai.ac.jp (Y.S.); 2 Unit of Risk Analysis and Management, Research Center for Zoonosis Control, Hokkaido University, Kita 20, Nishi 11, Kita-ku, Sapporo 001-00520, Hokkaido, Japan; satosito@ucm.es; 3 Global Station for Zoonosis Control, Global Institute for Collaborative Research and Education, Hokkaido University, Kita 20, Nishi 10, Kita-ku, Sapporo 001-0020, Hokkaido, Japan

**Keywords:** animal movements, bovine viral diarrhea virus, network analysis, PageRank, phylogenic tree, viral transmission

## Abstract

Bovine viral diarrhea (BVD) caused by BVD virus (BVDV) leads to economic loss worldwide. Cattle that are persistently infected (PI) with BVDV are known to play an important role in viral transmission in association with the animal movement, as they shed the virus during their lifetime. In this research, the “hot spot” for BVD transmission was estimated by combining phylogenetic and epidemiological analyses for PI cattle and cattle that lived together on BVDV affected farms in Tokachi district, Hokkaido prefecture, Japan. Viral isolates were genetically categorized into BVDV-1a, 1b, and 2a, based on the nucleotide sequence of the entire E2 region. In BVDV genotype 1, subgenotype b (BVDV-1b), cluster I was identified as the majority in Tokachi district. Network analysis indicated that 12 of the 15 affected farms had cattle movements from other facilities (PI-network) and farms affected with BVDV-1b cluster I consisted of a large network. It was implied that the number of cattle movements themselves would be a risk of BVD transmission, using the PageRank algorithm. Therefore, these results demonstrate that cattle movements would contribute to disease spread and the combination of virological and epidemiological analysis methods would be beneficial in determining possible virus transmission routes.

## 1. Introduction

Bovine viral diarrhea (BVD) is a systemic disease caused by the infection of the bovine viral diarrhea virus (BVDV), which belongs to Pestivirus A or B genus *Pestivirus*, family *Flaviviridae*, according to their genotype [1]. The virus possesses a single-strand positive-sense RNA of approximately 12.3 kb with one large open reading flame, flanked by a 5′ and 3′ untranslated region (UTR). The open reading frame encodes a single polyprotein, which yields four structural proteins (C, E^rns^, E1 and E2) and eight nonstructural proteins (N^pro^, p7, NS2, NS3, NS4A, NS4B, NS5A, NS5B) through co- and post-translational processing by cellular and viral proteases [2]. On the basis of the nucleotide sequence of the 5′UTR or the E2 gene, isolates are categorized into genotypes or subgenotypes. While the 5′UTR is the most conserved region in pestiviruses and is often used for viral detection for diagnosis, the E2 gene, which encodes a major target of neutralizing antibodies, is the most variable and used for comparative genomic analysis [3,4]. There are two types of BVDV infection: transient infection and persistent infection. When BVDV infects naïve adult cattle, they show mild clinical manifestations and are called transiently infected (TI) cattle, but they later develop antibodies within 2–3 weeks [5,6]. Alternatively, if naïve pregnant cattle are affected by the infectious BVDVs during the first 30–150 days of the pregnancy period, they would deliver persistently infected (PI) fetuses, which are immunotolerant with BVDVs and shed the virus throughout their life without showing any clinical manifestations [7,8]. Therefore, these PI cattle are considered spreaders of BVDV to other cattle that lived together with them in herds. BVD is listed as a notifiable disease in Japan because of its impact on the loss of cattle productivity. Once cattle are diagnosed as BVDV positive, a report to their local government is mandatory. The government should take appropriate control measures, including culling the PI cattle and undertaking additional investigations into the cattle housed together [9]. In addition to the passive notification in Tokachi district, active surveillance against BVD by using bulk tank milk samples is performed in Hokkaido prefecture, which is one of the biggest dairy milk production sites in Japan [10]. When PI cattle are detected using bulk tank milk samples, farmers are strongly advised to conduct a definitive diagnosis of PI cattle, as the diagnosis based on those samples cannot be replaced with the official one. PI cattle are culled following positive results of a definitive diagnosis, as it would take a long time to complete the entire steps of official diagnosis, accelerating disease spread within a farm. Therefore, farmers would adopt an option to cull the PI cattle spontaneously in the early stage, immediately after diagnosis using bulk tank milk samples. Even though those strict measures were enforced and the number of reported PI cattle had decreased, the emergence of PI cattle in Tokachi district remains continually reported [11], meaning that the achievement for complete eradication is long away. Although BVD is an eradicable disease through applying strict measures, as conducted in Scandinavian countries [12,13], a feasible and sustainable control scheme for BVD should be accepted as an alternative policy, by identifying risk factors for disease transmission and optimizing the cost-effectiveness of control measures [14,15].

Animal movement is commonly recognized as one of the risk factors of spreading BVD infection [16], while the relationship between animal movement and BVD infection has seldom been reported in Japan [17]. Given the transmission mode of BVDV, the presence of infected and sensitive individuals together in a certain space should be essential to establish disease transmission. Thereby, the movement of husbandry animals due to trade and shipment, which is attributed to economic activities, would heavily contribute to disease dynamics. The association between animal movements and disease spread is reported not only in bovine transmissive diseases, such as bovine tuberculosis [18], foot and mouth disease [19], and Johne’s disease [20], but also others, such as rabies [18] or porcine epidemical diarrhea virus [21]. Viral transmission is typically enhanced at a particular place with several risk factors. A farm with highly concentrated cattle access, revealed by tracing the cattle transmissive pathway, is called a “hot spot” for BVD transmission. These hot spots potentially contribute to amplification and scattering of pathogens, and are thus identified as the main target in disease control measures. A network analysis is commonly used to visually confirm connections and interactions, with or without directions, among the units in the population. In addition, scoring in a quantitative manner could make it possible to evaluate the BVD transmission risk in each unit, according to the type of facility, including farm, breeding farm, common pasture, and market. Therefore, the network analysis would be beneficial in investigating places at a high risk of viral transmission, or evaluating the appropriate control strategies [22].

This study aimed to reveal the BVDV transmission dynamics, which will provide beneficial findings for BVD control in Japan, where BVD is endemic. Genetic characteristics of viruses isolated from PI cattle were analyzed to identify the circulating viral prevalence. Moreover, the animal movement network among facilities was described based on the record of PI cattle, and cattle housed together with them. By integrating the information on phylogenetic analysis, the transmission routes of specific viruses were indicated, and the disease extension in the cluster was identified more specifically. Furthermore, network analysis constructed in this study revealed that a farm at high risk of virus introduction had many cattle movements. The combination of genetic and epidemiological information related to BVDV infection could provide wider and more detailed findings of disease transmission in livestock. 

## 2. Results

### 2.1. Phylogenetic Analysis

**Figure 1:**
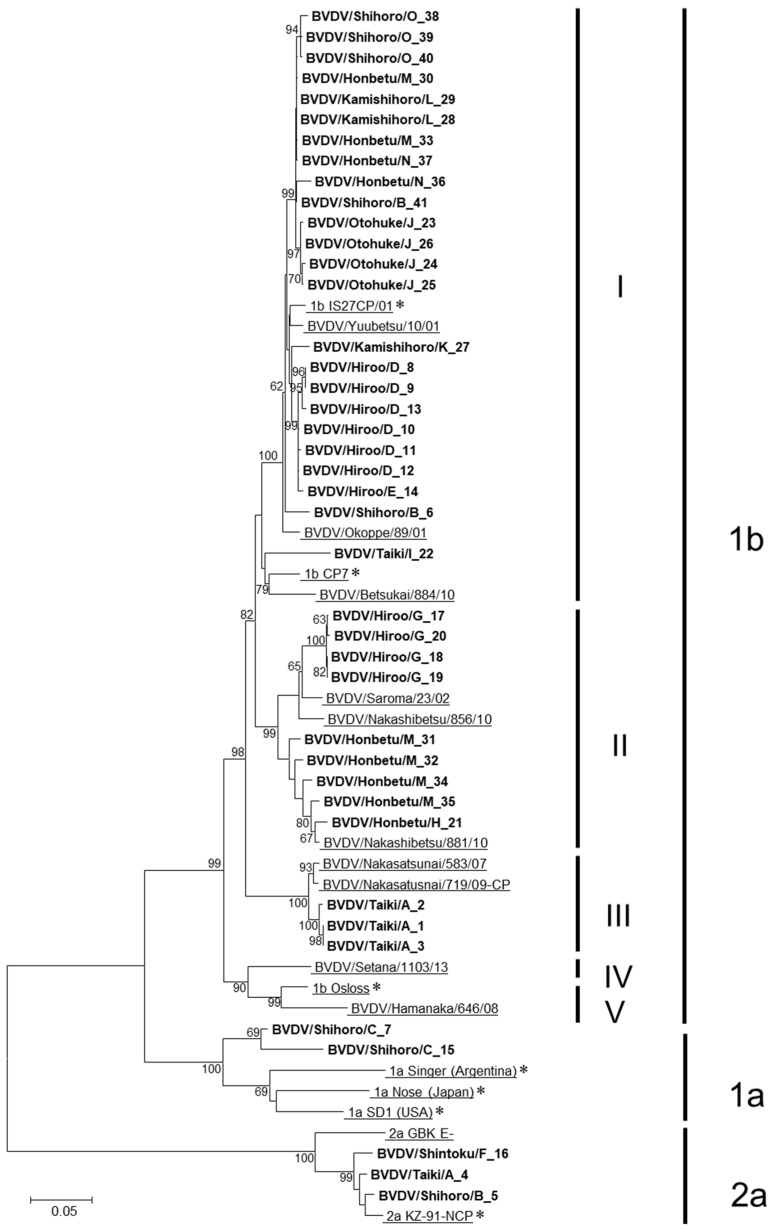
Phylogenetic tree of isolates based on the entire E2 gene. Nucleotides of the entire E2 gene (1122 bp) were used for genetic analysis. The phylogenetic tree was constructed using the neighbor-joining method and bootstrap analysis (*n* = 1000) on MEGA v.6.0 software. Bootstrap values (≥60) of phylogenetic analysis are shown in the phylogenetic tree. The isolated viruses in this study are named as farm BVDV/the name of isolated town/farm number_isolation number and indicated in bold. The reference strains for genotype and cluster classification are described with underlines, and an asterisk (*) is added behind the name of each strain, which was used for genotyping.

A total of 15 BVD-affected farms kindly provided 41 PI cattle serum samples and the ear tag numbers of 9395 cattle for individual identification, including them and other cattle housed together (Table 1). In the phylogenetic analysis, based on the entire E2 nucleotide sequences, BVDV isolates fell into the cluster of either BVDV-1 subgenotype 1a or 1b, or BVDV-2 subgenotype 2a (Figure 1). The isolates from the BVDV-1 subgenotype 1b (BVDV-1b) were further categorized into five clusters that were proposed in a previous study [23]. Most isolates were BVDV-1b (36 strains), particularly cluster I (23 strains). The prevalence of the BVDV genotype isolated in this study corresponded to isolates in Hokkaido prefecture from 2001 to 2014, which was previously reported [23], indicating that BVDV-1b, cluster I was the major group of circulating viruses in Tokachi since the beginning of the 21st century.

The number of BVDV-1a and 2a isolates was only 2 and 3, respectively. Those isolates that fell into the BVDV-1a strain were close to the Nose strain, which was isolated in Japan [24], whereas the BVDV subgenotype 2a (BVDV-2a) isolates were closely related to the KZ-91-NCP strain. This tendency is consistent with previous reports [23], therefore, it can be assumed that BVDVs found in Hokkaido prefecture were maintained within that area, and no new strain was sustained following introduction, in at least the past 20 years.

Most of the BVDV-1b isolates from the same farm were categorized into the same cluster. However, the viruses isolated in Farm M were classified into clusters I and II, implying that these viruses would be introduced to Farm M through at least two distinct routes.

The sequence identities among isolates within-herd and between-herds were ranged between 94.9–100% and 90.6–99.7%, respectively, which was calculated in each of the subgenotypes and clusters, particularly 1bs.

### 2.2. Network Analysis of Cattle Movement

**Figure 2:**
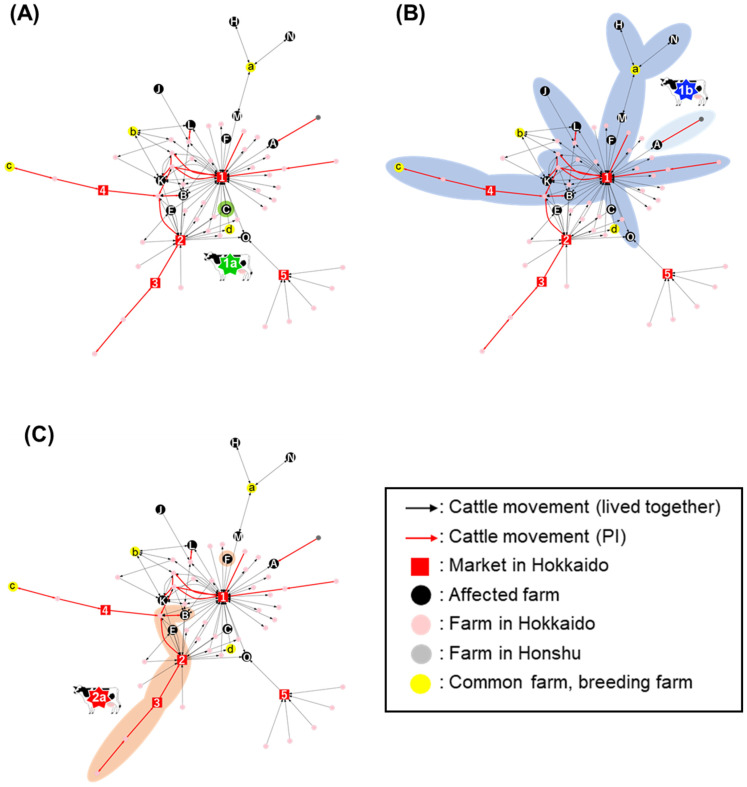
Cattle movements between affected farms overlaid with genotypes of virus isolates. The network was described on the basis of the animal movements acquired from the individual ear tag number of cattle, which focused on the cattle movements of PI cattle that passed more than two affected farms. Sixty-three facilities were included in the network, and 1200 cattle movements with 9 PI cattle movements were involved. Black and red arrows indicate the cattle movements of cattle that lived together with PI and of PI, respectively. Square nodes describe markets, whereas circular nodes represent farms. Each color indicates their type. The green, blue, and red colors overlaid on the network show the relationship between farms affected with BVDV 1a (**A**), 1b (**B**), and 2a (**C**), respectively. The association of BVDV-1b was further differentiated by dark and light blue, according to its cluster (dark blue shows cluster I, and light blue shows cluster III).

For the 15 BVD-affected farms, a network plot was described based on movements of the PI cattle and the cattle living with them at their detection, to visualize the hot spot by converting the cattle movements into farm connections. Then, the movement of PI cattle and cattle that connected affected farms were selected, and the network was reconstructed to form a PI network. The PI network contained 12 of the 15 affected farms, indicating that these farms had cattle movements regardless of the infectious status of cattle (Figure 2). The overlaid colors of the PI network indicated the classification of BVDV subgenotype or cluster in which the farm was affected as follows: dark blue, BVDV-1b cluster I; light blue, BVDV-1b cluster III; green, BVDV-1a; red, BVDV-2a. According to this network, the viruses that fell into the BVDV-1b cluster III originated from a farm in Honshu, Japan’s main island, and then transferred to a farm in Tokachi district, indicating that viruses categorized in BVDV-1b cluster III would be introduced from the main island of Japan. Movements of cattle infected with the BVDV subgenotype 1a and 2a were then further confirmed using the same network, although sporadically. One PI cattle infected with the BVDV-2a was transported across several farms and markets, without causing further virus transmission. In contrast with those isolates described above, cattle and farms affected with BVDV-1b cluster I viruses composed a wide network. Three PI cattle carrying the BVDV-1b cluster I virus were moved from facilities and were mediated by Market 1 in Tokachi district. Interestingly, five other affected farms introduced cattle, but the non-PI cattle originated from Market 1.

### 2.3. Centrality Analysis in the Network

#### 2.3.1. PageRank Analysis against the PI-Network (PI-PageRank)

In this study, the degree of concentration of cattle movements were expressed as the centrality of the network, using the PageRank algorithm. According to the results of the PI PageRank, in which only the cattle movements of PI-network were included, Market 1 ranked the second highest among all facilities in that extracted network (Table 2). All affected farms that ranked in the top 10 had introduced cattle from Market 1. A total of five markets in Hokkaido prefecture were included in the PI-network. Of the 63 facilities that comprised the PI-network, 2 markets within Tokachi district ranked in the top 10, and the others placed in Hokkaido prefecture but outside of the Tokachi district ranked 15th, 27th, and 46th. Taken together, cattle movements in Tokachi district tended to assemble at markets, especially Market 1, regarding the cattle movements between affected farms. These results support the importance of cattle movements as a risk of BVD transmission, and the assumption that the market was implicated as the hot spot for BVD transmission in Tokachi district.

#### 2.3.2. Association between PI PageRank and the PageRank Based on All Farm Connections

**Figure 3:**
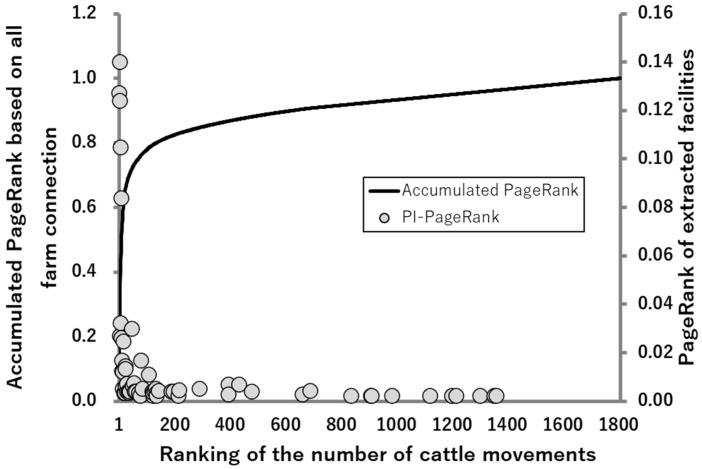
The relationship between PI PageRank and PageRank based on all farm connections. The PageRank algorithm was applied to both the PI-network and the network of all farm connections. The calculated PageRank values of each facility included in the PI-network are shown as dots, and the results against all farm connections were accumulated from the top and are indicated as a solid line.

A PageRank analysis was also applied to all the facilities (totally 1809 farms, markets, common pastures, or breeding farms) based on all cattle movements. The algorithm was accumulated from the top, and depicted as a solid line in Figure 3. The PageRank scores of the farms and markets were also included in the PI-network, that is, 63 facilities, were overlayed as dots. The accumulated PageRank demonstrated that 80% of all cattle movements into Tokachi district were counted in the top 135 of 1809 facilities (7.5%). Among the top 135 facilities, 40 facilities were included in the PI-network, which makes up 63.5% of the facilities that constituted the extracted network. The other 36.5% of the facilities in the extracted network were included in 92.5% of the facilities with less movement. These results propose that more frequent cattle movements are likely to lead to the emergence of PI cattle, either by direct introduction of PI or secondary infection from PI cattle.

## 3. Discussion

Phylogenetic analysis on the basis of the whole E2 gene indicated that BVDV isolates in Tokachi district were classified into BVDV-1a, 1b, and 2a strains. These viruses and the BVDV subgenotype 1c were reported to circulate since 2001 at the earliest in Hokkaido prefecture, Japan [23,25]. Among them, the BVDV-1b viruses remain predominant. The BVDV-1b field isolates were unlikely to show typical clinical signs in cattle [23], thus resulting in the high prevalence and frequency of isolates in Tokachi district. On the other hand, the number of BVDV-1a isolates decreased between 2000 and 2014 [23]. In Japan, vaccination is the basic control measure against BVD, with inactivated vaccines or live-attenuated vaccines being assumed to contain at least the BVDV-1a antigen [9]. The decline in BVDV-1a infections would support the success of vaccination, whereas various phylogenetic differences among BVDV-1b compared with BVDV-1a isolates lead to different results in vaccination policy.

In this study, cattle in the 12 affected farms were introduced from outside, and their movement pathways formed the overlapped network. Furthermore, 80% of the cattle movements in this study were performed by 7.5% of the high-risk facilities, which were 63.5% of farms that made up the PI-network. These results imply that the emergence of PI cattle should be attributed to cattle movements, and occurs in farms with more frequent cattle movements; this is in agreement with the previous report [26]. In that report, the effect of virus transmission through contact at markets was ignored. However, the authors mentioned that those contacts could have a larger impact, suggesting that markets should be involved in control measures [26]. Furthermore, the most common way of BVDV transmission was reported as the movement of PI cattle between herds [27], which was attributed to at least some cases in Switzerland [28]. In a prefecture in Honshu, Japan, the PI cattle occurrence was also proportionally reported to correlate with the rate of cattle purchased [29].

According to the results that most of the cattle movement in Tokachi district was localized to only 7.5% of facilities obtained in this study, contrary to the emergence and expansion of PI cattle, a large number of total cattle movements were limited to certain farms and transmission pathways. This increase in the number of PI cattle movements occurred at a fixed proportion. In this study, 12 of the 15 (80%) affected farms introduced cattle from outside, and the routes of the affected PI cattle partially overlapped with each other, as they were plotted on the same network. However, the relatively higher proportion of PI cattle that was in small farms, such as Farm L, would pose other conceivable factors, such as contributing to the emergence of PI cattle in addition to the number of cattle movements. Each facility has a different risk of BVD transmission, as shown by the PageRank algorithm; therefore, the mediated venue is also important for regional disease transmission. This means that cattle, even on a farm with cattle movements in few numbers, would potentially be at a high risk by passing the high-risk facilities. A previous report demonstrated that the high frequency of animal movements between certain farms was a risk of BVD, which supports our results of the PageRank analysis [30].

All of the PI cattle infected with the BVDV-1b cluster I, which passed the same Market 1 in Tokachi district, and eight affected farms had a connection to Market 1 in the PI-network. Concentrations of cattle movements in the PI-network plot gathered to this market, and the PI PageRank algorithm as a numerical method implied that Market 1 should be the hot spot for BVD transmission in this region. All the cattle introduced to markets were neither inspected for viral infection nor received a confirmation of vaccination history. As BVDV was transmitted from one cattle to another within 24 h [31,32], these facts imply that markets, where many cattle were kept together within close distances, were the high-risk facilities for horizontal transmission. Meanwhile, in the other affected farms, all cattle were kept on their farms. BVD spread on the farms and led to the delivery of immunotolerant fetuses on the farms, along with PI cattle movements, whereas about 80% of the PI cattle were born and had stayed in their birthplace until they were detected. Other factors, such as virus introduction by TI cattle or machinery transmission through humans and vehicles can be considered but were not validated in this study.

In Farm M, no PI cattle movements were observed, whereas there were frequent cattle movements within a common pasture. In Farms H and N, which do not have PI cattle movements, cattle movements were limited only within common pastures, whereas isolated viruses were classified into the same genotype and cluster, the BVDV-1b cluster I. This implies that BVDV can be introduced from common pastures and maintained within a farm. It is also considered that some cattle were transiently infected with BVDV in a common pasture, and they then gave birth to PI cattle via transplacental infection, thus leading to the generation of PI cattle and further spread of BVDV after the return to their original farms [29,33]. All of the PI cattle, except one in this study, were neither moved into nor moved out of the common pasture. In Japan, an acceptance inspection of BVD and other transmissive diseases was said to be performed at common pastures before cattle entered the farm. This inspection may contribute to the prevention of the PI cattle introduction to common pastures. However, it is probable that the cattle at the latent period of the BVDV infection passed the acceptance inspection due to being under the detection limit of diagnosis, and shed the infectious viruses within the farms to cause the horizontal infection to other cattle. Although this possibility might be low compared with PI cattle, as the latent period was less than one week, TI cattle could be the source of infection given that there were many cattle in the common pasture. It was reported that the transient infection of cattle in common pastures can be one of the BVDV infection routes, in addition to PI cattle introduction [33,34]. Therefore, not only PI but also TI cattle should be considered to play a key role in BVD transmission.

Some markets have already taken voluntary preventive measures, such as confirmation of prior vaccination history; nevertheless, the market is identified as one of the hot spots of BVDV spread in this region. As the risk of PI cattle emergence is low in common pastures, an acceptance inspection of introduced cattle would be effective for preventing BVD introduction, and is preferred to be implemented in markets as well. Moreover, BVD is introduced to a farm not only by PI cattle movements but also by TI cattle movements, and, therefore, the combined strategy of acceptance inspection and vaccination could be effective for BVD eradication in Japan. We should also understand the risk of disease spread by animal movements and protect healthy cattle from the threat of PI cattle, through appropriate preventive measures, such as inspection and culling or vaccination. The government should also improve its control strategy for comprehensive prevention against BVDV on a regional scale.

## 4. Materials and Methods

### 4.1. Characteristics of the Farms and Animals in This Study

Between 2018 and 2020, the local government office in Tokachi district, Hokkaido prefecture, Japan received BVDV notifications in 80 farms total, including suspected cases [11]. Among the 9395 cattle in 15 of 80 farms, which were named A to O, 41 PI cattle were detected (Supplementary Table S1). Information regarding the farm size and classification of BVDV isolates on the affected farms is presented in Table 1. Various farm sizes were observed, ranging from less than 80 to more than 4000 cattle. The local government office in Tokachi district performed the definitive diagnosis of PI cattle, following the positive results in the bulk tank milk sample test. Then, they investigated the BVDV infections for all cattle housed together in a farm when PI cattle were officially identified. With consent, animal owners kindly provided the serum samples of these isolates and the individual identification numbers of all cattle housed together.

### 4.2. Phylogenetic Analysis

#### 4.2.1. Viruses and Cells

Viruses were isolated from cattle or fetuses’ serum following positive results from the reverse transcription polymerase chain reaction (RT-PCR) against BVDV 5′UTR with the primer set 324 and 326, as described previously [23,25,35], and all isolates were noncytopathogenic. Isolates were then named as “BVDV/the name of the isolated town/farm number_isolation number”. For example, “BVDV/Taiki/Farm A_1” represented the virus that was isolated in Farm A, Taiki town in Tokachi district. Those strains were initially isolated from the Madin-Darby bovine kidney (MDBK) cell line and were propagated in Eagle’s minimum essential medium (Nissui Pharmaceutical, Tokyo, Japan) supplemented with a 0.295% tryptose phosphate broth (Becton Dickinson, San Jose, CA, USA), 10 mM *N, N-bis* (2-hydroxyethyl)-2-aminoethanesulfonic acid (Sigma-Aldrich, St. Louis, MO, USA), 0.3 mg/ml L-glutamine (MEM-BES), and 10% horse serum (Life Technologies, Carlsbad, CA, USA), consecutively. Serum samples were inoculated into MDBK cells within 72–96 h of incubation, and viral growth in the cell was confirmed by immunostaining, as described previously [36]. Cells inoculated with viruses were then air-dried and fixed at 80 °C for 1 h. The cells were also stained with anti-NS3 MAbs [37]. Thereafter, the supernatant of the cell culture was further used for the genetic analysis.

#### 4.2.2. RT-PCR and Sequencing

Viral RNA was extracted using the TRIzol LS Reagent (Life Technologies) from the supernatants of MDBK cells infected with viruses. For amplification of the whole E2 gene, the extracted RNA was reverse-transcribed with a Random Primer (N)9 (Takara Bio, Otsu, Japan). The Reverse Transcriptase SuperScript III (Thermo Fisher Scientific Inc., Waltham, MA, USA) and primers were used according to a previous report [23]. After viral RNA amplification with KOD Fx Neo (TOYOBO, Osaka, Japan), the BigDye Terminator v3.1 Cycle Sequencing Kit (Life Technologies), and the 3500 Genetic Analyzer (Life Technologies) were used to determine the nucleotide sequences of PCR fragments according to the manufacturer’s protocol. Sequencing data were also analyzed using the GENETYX Network version 15 software (GENETYX, Tokyo, Japan).

#### 4.2.3. Phylogenetic Tree

Multiple sequences were aligned using the CLUSTAL W algorithm, using the default parameters for phylogenetic analysis [16,38]. A phylogenetic tree based on the nucleotide sequences of whole E2 genes was also constructed by a neighbor-joining method and bootstrap analysis (*n* = 1000), using MEGA 6.0 software with default parameters [39]. This contained the sequence data of reference strains that were obtained from the DDBJ/EMBL/GenBank database, whereas those of BVDVs were isolated from 2001 to 2014 as described in previous reports [23,25]. BVDVs of Nose (BVDV-1a; AB019670), IS27CP/01 (BVDV-1b; AB359935), KZ-91-NCP (BVDV-2a), Hokudai-Lab/09 (BVDV-2b; AB567658), and GBK_E^–^ (BVDV-2a; AB894424) were selected as reference strains for genotyping [23,24,40]. BVDV/Yuubetsu/10/01 (AB896805), BVDV/Okoppe/89/01 (AB896807), BVDV/Betsukai/884/10 (AB896811), BVDV/Nakashibetsu/856/10 (AB896810), BVDV/Nakashibetsu/881/10 (AB894349), BVDV/Saroma/23/02 (AB896806), BVDV/Nakasatsunai/719/09-CP (AB896809), and BVDV/Nakasatsunai/583/07 (AB896808) [23] were also selected as reference strains in the phylogenetic analysis for further classification of the cluster of BVDV-1b isolates.

### 4.3. Epidemiological Analysis

#### 4.3.1. Network Analysis

Network analysis was conducted in R (R foundation), using the igraph package (v.1.2.5) [41]. A farm-level network was established to represent the connectivity between sites through cattle movements, using circle and square nodes to represent farms and markets, respectively, and with arrows representing the movement of PI cattle and cattle living with them using the anonymized animal-movement data (Supplementary Table S2). For example, self-loops, which are movements within the same farm, were removed. Multiple movements between the same sites were also integrated to form a single connection, and arrows within the network were non-weighted. Movements occurring in different directions, for example, Farm A to B and Farm B to A, were differentiated in this network. After the construction of a farm-level network, the genotypes of isolates from each affected farm were indicated manually, by using different colors with Microsoft Office PowerPoint.

#### 4.3.2. PageRank Analysis

The PageRank algorithm is one of the methods of assessing the centrality of the network in a target population, and is used for a directed network, which has difficulty detecting the eigen vector centrality [42]. Movements of cattle were obtained, and directionality between farms were displayed in an adjacency matrix and then a transition probability matrix, which indicates the probabilities of animal movements from each farm to connected farms. The transition probability matrix was adjusted to convert it into an irreducible matrix, to introduce the formula below which enables the calculation of the highest-magnitude eigenvector. The adjusted transition probability matrix, *M*’, was then obtained using the following formula:

$$M^{\prime }=cM+\left(1-c\right){\left[\frac{1}{n}\right]}_{\;\;n\times n},$$

where *c* is called a damping factor and is set to 0.85 in this study. *n* is the number of farms in the population, which is equal to the row (and column as well) number of irreducible matrices. [1/*n*] *_n_*_×*n*_ is a *n* × *n* square matrix, in which all the elements are 1/*n*. The first eigen value of *M*’ is obtained as the scores of the PageRank algorithm in each farm. All network analyses and PageRank algorithms were performed using the R program version 3.6.1 (R foundation).

## Figures and Tables

**Table 1:** Characteristics of BVDV affected farms in Tokachi between 2018 to 2020.

	Number of Cattle	Number of PI Cattle	Isolates of Each Genotype		
			1a	1b	2a
A	608	4	0	3	1
B	605	3	0	2	1
C	1693	2	2	0	0
D	118	6	0	6	0
E	613	1	0	1	0
F	30	1	0	0	1
G	110	4	0	4	0
H	83	1	0	1	0
I	107	1	0	1	0
J	128	4	0	4	0
K	4120	1	0	1	0
L	78	2	0	2	0
M	295	6	0	6	0
N	84	2	0	2	0
O	723	3	0	3	0
Total	9395	41	2	36	3

**Table 2:** Top 10 farms and facilities of the PageRank in the PI network (PI-PageRank).

Ranking in the PI Network	PI-PageRank	Facility Status
1	0.140	Farm K
2	0.128	Market 1
3	0.124	Farm in Hokkaido
4	0.105	Common farm a
5	0.084	Farm A
6	0.032	Market 2
7	0.030	Breeding farm b
8	0.027	Farm O
9	0.026	Farm B
10	0.025	Farm in Hokkaido

## Data Availability

All the data used in this study was kindly provided by the farmers in Tokachi District, Hokkaido, and the data used in the network analysis were available from the Supplementary Materials after anonymization.

## Supplementary Materials

The following are available online at https://www.mdpi.com/article/10.3390/pathogens10080922/s1, Table S1: Information of 41 BVDV field isolates in Tokachi district, Hokkaido prefecture, Japan in this study, Table S2: De-identified data of cattle movements used for network analysis and PageRank analysis.
